# Changed Expression of Cytoskeleton Proteins During Lung Injury in a Mouse Model of *Streptococcus pneumoniae* Infection

**DOI:** 10.3389/fmicb.2018.00928

**Published:** 2018-05-08

**Authors:** Mario Ferrer-Navarro, Anja Strehlitz, Eva Medina, Jordi Vila

**Affiliations:** ^1^Instituto Salud Global, Barcelona Centre, International Health Research, Hospital Clínic—Universitat de Barcelona, Barcelona, Spain; ^2^Infection Immunology Research Group, Helmholtz Centre for Infection Research, Braunschweig, Germany

**Keywords:** *Streptococcus pneumoniae*, pneumonia, lung injury, DIGE, cytoskeleton

## Abstract

Infections by *Streptococcus pneumoniae* are a major cause of morbidity and mortality worldwide, often causing community-acquired pneumonia, otitis media and also bacteremia and meningitis. Studies on *S. pneumoniae* are mainly focused on its virulence or capacity to evade the host immune system, but little is known about the injury caused in lungs during a pneumococcal infection. Herein we investigated this issue comparing the proteome profile of lungs from *S. pneumoniae-*infected mice with control mice by means of difference gel electrophoresis (DIGE) technology. In order to obtain reliable results three biological replicas were used, and four technical replicas were carried out in each biological replica. Proteomic comparison was performed at two time points: 24 and 48 h post infection. A total of 91 proteins were identified with different abundance. We found important changes in the protein profiles during pneumococcal infection mainly associated with regulation of vesicle-mediated transport, wound healing, and cytoskeleton organization. In conclusion, the results obtained show that the cytoskeleton of the host cell is modified in *S. pneumoniae* infection.

## Introduction

Since the isolation of *Streptococcus pneumoniae* in 1881 these bacteria have been one of the most extensively studied human pathogens. Despite the numerous studies on *S. pneumoniae* many questions regarding its virulence and the effect of pneumococcal infection on the lungs remain unanswered (Johnston, 1991; Kadioglu et al., 2008). As a human pathogen, *S. pneumoniae* is the most common bacterial cause of acute respiratory infection, especially community-acquired pneumonia. Indeed, this pathogen causes over 3 million deaths in children worldwide every year by infections such as pneumonia, bacteremia, and meningitis (Greenwood, 1999). Until recently, pneumococcal infections could be effectively treated with penicillin, an inexpensive and safe antibiotic. However, the spread of penicillin-resistant pneumococci has been on the rise (Cillóniz et al., 2016), with the consequent increase in treatment costs and the need for more expensive antimicrobial drugs which may be beyond the reach of the health services of poor, developing countries. Since the introduction of polysaccharide-based vaccines the number of severe cases of pneumococcal infection has greatly decreased in developed countries. However, the use of vaccines in poor, developing countries requires substantial investment, not only in vaccine supplies but also in system costs, such as cold chain expansions (Griffiths et al., 2016). The phenomenon of serotype replacement after the introduction of conjugate pneumococcal vaccines and the rise in antibiotic resistance worldwide continues to be an important issue in terms of the impact on clinical outcomes in pneumococcal disease. Studies on *S. pneumoniae* are mainly focused on its capacity to evade the immune system (Aguinagalde et al., 2015; Rai et al., 2016), its virulence (Kadioglu et al., 2008; Brown et al., 2016), and the search for potential vaccine components (Choi et al., 2016; Ochs et al., 2016).

In recent years, innovative proteomic methods combining high resolution with high throughput in the search for biomarkers of disease have evolved and improved, with different proteomic technologies and strategies being applied according to the study undertaken (Tirumalai et al., 2003; Jacobs et al., 2005; Schiess et al., 2009). In principal two different strategies are applied for the analysis of a particular proteins sample. These are known as Bottom-up and Top-down proteomics. The Bottom-up approach begins with the enzymatic digestion of proteins into peptides that are then separated, identified, or quantified by mass spectrometry. This approach allows the identification of a huge number of different peptides, but the main challenge is the high complexity of protein mixture that becomes extremely complex by digestion into peptides. Furthermore, information about different protein isoforms as well as post-translational modification (PTM) is lost with this approach. In contrast, in the Top-down approach proteins are separated intact, and thus, neither isoform nor PTM information is lost. While two-dimensional electrophoresis has several advantages it also has some drawbacks: proteins with extreme p*I* or molecular mass are poorly represented. In addition, the separation of hydrophobic or membrane proteins as well as alkaline proteins remains difficult.

All in all proteomics is a powerful tool for studying infectious diseases and integrative “omics” approaches can provide understanding of the host-pathogen interactions (Jean Beltran et al., 2017). Different studies have employed different proteomic approaches in order to identify changes in protein abundance in the host during infection. For example, Díaz-Pascual et al. used proteomics approaches to elucidate the effect of *Pseudomonas aeruginosa* in zebrafish as an animal model (Díaz-Pascual et al., 2017). Méndez et al. employed quantitative proteomics in order to characterize the proteome of *Acinetobacter baumanii* under conditions that simulate those found in the airways (Méndez et al., 2015). Seddigh et al. recently studied the effect of *Aspergillus fumigatus* infection on alveolar epithelial cells by means of proteomic approaches (Seddigh et al., 2017).

In the present study, we used a murine model of *S. pneumoniae* infection to evaluate the underlying differences in protein expression in the lungs of *S. pneumoniae*-infected mice by means of fluorescence-based two-dimensional difference gel electrophoresis (DIGE) (Görg et al., 2004). To carry out this study we used the TIGR4 strain of *S. pneumoniae*. This strain is a highly virulent capsular serotype 4 clinical isolate obtained from the blood of a 30-year-old male patient in Kongsvinger, Norway (Aaberge et al., 1995). The genome of this strain was described in Tettelin et al. (2001). The TIGR4 isolate was previously reported as JNR.7/87, the label of the clinical isolate (Bricker and Camilli, 1999), but it can also be found in the literature as KNR.7/87 (de Saizieu et al., 2000); and as N4 (Wizemann et al., 2001).

## Materials and methods

### Culture of *S. pneumoniae*

The *S. pneumoniae* serotype 4 strain TIGR4 was grown at 37°C in Todd-Hewitt broth (Sigma-Aldrich, Munich, Germany) supplemented with 1% (w/v) yeast extract (Sigma-Aldrich) and 1% of heat-inactivated fetal calf serum (GIBCO/Invitrogen, Eggenstein-Leopoldshafen, Germany). Bacteria were grown to the Mid-Log phase (OD_600_), collected by centrifugation for 10 min at 4,000 rpm, and washed once with sterile phosphate-buffered saline (PBS). For the inoculum, the bacterial suspension was diluted with PBS to the concentration required.

### Animals and infection model

Female Balb/c mice were purchased from Envigo (Horst, Netherlands) and kept under conventional conditions with *ad libitum* access to food and water. Mice of 10–12 weeks of age were used in all experiments. The mice were anesthetized by intraperitonal injection with ketamine 10% (100 mg/kg; Wirtschaftsgenossenschaft deutscher Tierärztee eG, Garbsen, Germany) and xylazine 2% (10 mg/kg; CP-Pharma GmBH, Burgdorf, Germany) and intranasally inoculated with either 5 × 10^7^ colony forming units (CFU) of *S. pneumoniae* in 20 μl of PBS or with mock treatment with sterile 20 μl PBS. The mice were weighed after 24 and 48 h. The animals were euthanized by CO_2_ asphyxiation, bacterial loads were determined in the lungs and systemic organs. Blood was collected and supplemented with ethylenediaminetetraacetic acid (EDTA) and measured in the hematologic system VetScan HM5 (Abaxis). Absolute cell counts for each population were used to determine the ratio of granulocytes to lymphocytes in blood.

### Sample preparation and proteome analysis of lung tissue

For proteome analysis, the lung tissue was washed in PBS after collection, patted with tissue paper, cut into appropriate pieces, placed into pre-labeled tubes and snap-frozen in liquid nitrogen. In total, three biological replicates per condition and time point of the lung tissue of 3 mice each were obtained. The samples were stored at −80°C until sample preparation.

The lung tissue samples were homogenized in lysis buffer (7 M urea, 2 M thiourea, 5% 3-[(3-cholamidopropyl) dimethylammonio]-2-hydroxy-1-propanesulfonate, 5% 3-[N,N-Dimethyl(3-myristoylaminopropyl)ammonio]propanesulfonate supplemented with protease inhibitor (GE Healthcare) by filtering through a 40 μm cell strainer. The homogenate was sonicated for 10 min with 1 min breaks at 4°C in ice-water and then centrifuged at 17,000 × g for 5 min to pellet the remaining debris. The supernatant was aliquoted and stored at −80°C. The protein extracts were quantified with the 2-D Quant Kit and treated with the 2-D Clean-up Kit. A protein concentration of 50 μg of each sample was minimally labeled with 400 pmol CyDyes DIGE Fluors: Cy3 for PBS controls and Cy5 for *S. pneumoniae* TIGR4 infected samples and vice versa to reduce variation due to labeling. An internal standard was generated by combining equal amounts of extracts from all the samples generated at 24 or 48 h, respectively, and labeled with Cy2. The samples were incubated with the CyDyes for 30 min on ice in the dark at pH 8.5. One micro liter of 10 mM lysine was added to quench the reaction and the samples were then incubated for 10 additional minutes on ice in the dark. After labeling, immobilized pH gradient buffer 3-10 at a final concentration of 0.5% was added to the samples as well as dithiothreitol (DTT) at a final concentration of 20 mM, and finally, Destreak reagent (GE Healthcare), was used for IPG Strip hydration following the manufacturer's recommendations.

We performed four technical replicas of each biological replica. A total of 4 DIGE gels of each biological replica and per time point were run, separating two randomly picked samples of PBS control and *S. pneumoniae* TIGR4-infected animals. The labeled mixtures were combined, together with the internal standard and loaded onto a Immobiline Dry Strip (3–10 pH, 24 cm) for first dimension separation by isoelectric focusing for 19 h (200 V for 2 h, 500 V for 2 h, 1,000 V for 4 h, and 8,000 V for 11 h, 20°C) in a IPGphor I (GE Healthcare). After focusing, each strip was equilibrated for 15 min in 10 mL of SDS equilibration buffer solution (6 M Urea, 75 mM Tris-HCl pH 8.8, 29.3% glycerol, 2% SDS and grains of bromophenol blue) supplemented with DTT or iodoacetamide, respectively, loaded on a precast 12.5% polyacrylamide gel and run using an Ettan DALTsix Electrophoresis System (GE Healthcare) at the following settings: 80 V, 10 mA/strip, 1 w/strip for 1 h, and 500 V, 40 mA/strip, 13 w/strip until the bromophenol blue tracking front ran off the end of the gel. Fluorescence images of the gels were obtained on a Typhoon 9400 scanner (GE Healthcare). Cy2, Cy3, and Cy5 images were scanned at excitation/emission wavelengths of 488/520, 532/580, and 633/670 nm, respectively, at a resolution of 100 μm. Image analysis was performed using Progenesis SameSpots 4.6.206 (Totallab, Newcastle, UK) as well as molecular weight and p*I* estimation according to the software manufacturer's guidelines and manual.

### Gel staining and detection of proteins

In order to visualize spots, 2-D gels were silver stained as described elsewhere (Párraga-Niño et al., 2012). Briefly, the gels were fixed twice in 40% ethanol and 10% acetic acid for 30 min each. Sensitizing was carried out for 30 min in 0.02% (w/v) sodium thiosulfate. The gels were then washed three times for 5 min with distilled water and incubated in 0.1% (w/v) silver nitrate for 20 min. The gels were washed again twice for 1 min with distilled water and developed with 3% (w/v) sodium carbonate and 0.025% (v/v) formaldehyde until the desired contrast was reached. Reaction was stopped with 1.5% (w/v) EDTA-Na_2_ for 45 min, after which the gels were washed twice with distilled water.

### In-gel tryptic digestion

In-gel trypsin digestion was performed as described previously (Shevchenko et al., 1996) from silver stained gels. Selected spots were excised from 2D gels using a cut tip and silver was washed out from the spots with 200 μL of 30 mM potassium ferricyanide and 100 mM sodium thiosulfate (1:1) for 20 min in the dark. The spots were then washed with Milli-Q water until completely clear. Before tryptic digestion, reduction, and alkylation with DTT/iodoacetic acid (IAA) was performed by incubating the samples with 200 μL of 10 mM DTT in 50 mM ammonium bicarbonate for 1 h at 56°C, followed by alkylation with 200 μL of 55 mM IAA in 50 mM ammonium bicarbonate for 30 min at room temperature, protected from light. Gel pieces were digested overnight with 6 ng/μL trypsin at 37°C. The peptide extraction was carried out with three consecutive washes with 0.2% trifluoroacetic acid. The eluted peptides were dried in a SpeedVac and stored at −20°C until analysis by mass spectrometry.

### Mass-spectrometry analysis

For matrix-assisted laser desorption ionization (MALDI) analysis, spot digestions were desalted using ZipTips C_18_, and 1 μL of sample was mixed with the same volume of a saturated solution of α-cyano-4-hydroxy-trans-cinnamic acid matrix (0.5 mg/mL in acetonitrile/water/TFA 1% 3:6:1) and spotted onto a MALDI target plate (Bruker). The drop was air-dried at room temperature. MALDI-mass spectra were recorded in the positive ion mode on an UltrafleXtreme™ time-of-flight instrument (Bruker). Ion acceleration was set to 25 kV. All mass spectra were externally calibrated using a standard peptide mixture containing angiotensin II (1046.54), angiotensin I (1296.68), substance P (1347.74), bombesin (1619.82), renin substrate (1758.93), adrenocorticotropic hormone 1-17 (2093.09), adrenocorticotropic hormone 18-39 (2465.20), and somatostatin 28 (3147.47). Calibration was considered good when a value below 1 ppm was obtained. For peptide mass fingerprinting analysis, the Mascot search engine (Matrix Science) was used with the following parameters: one missed cleavage permission site, 100 ppm measurement tolerance, and at least four matching peptide masses. Cysteine carbamidomethylation was set as a fixed modification when appropriate, with methionine oxidation as the modification variable. First, common contaminants were removed using the contaminants database available in the Mascot search engine, and then searches were performed without taxonomy restriction using the NCBInr database (release 79). Positive identifications were accepted with a Mascot score corresponding to a *p*-value ≤ 0.05.

### Statistical analysis

Statistical analyses were carried out using GraphPad Prism 5.01 (GraphPad Software, San Diego, CA). The data are expressed as mean ± SD. Comparison between groups or time points was made using the two-tailed Student's *t-*test or one-way ANOVA in GraphPad Prism. Statistical quantification of relative protein levels was performed with Progenesis SameSpots 4.6.206 (Totallab, Newcastle, UK).

## Results and discussion

### Characterization of the pneumonia mouse model

**Figure 1:**
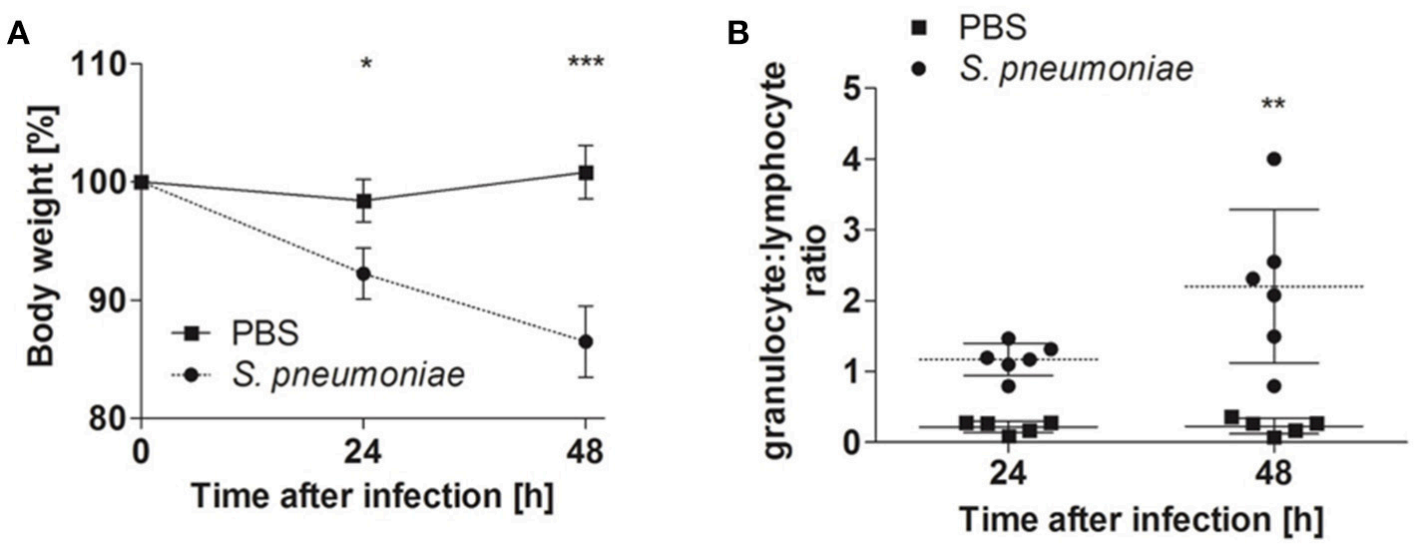
Body weight and blood granulocyte/lymphocyte ratios of Balb/c mice intranasally inoculated with either *S. pneumoniae* or treated with PBS. Balb/c mice were intranasally inoculated with 5 × 10^7^ of *S. pneumoniae* (circle) or PBS (rectangle), and the body weight **(A)**, and blood granulocyte/lymphocyte ratio **(B)** were determined at 24 and 48 h of infection. One representative experiment out of three is shown *p < 0.05; **p < 0.005; ***p < 0.001.

**Figure 2:**
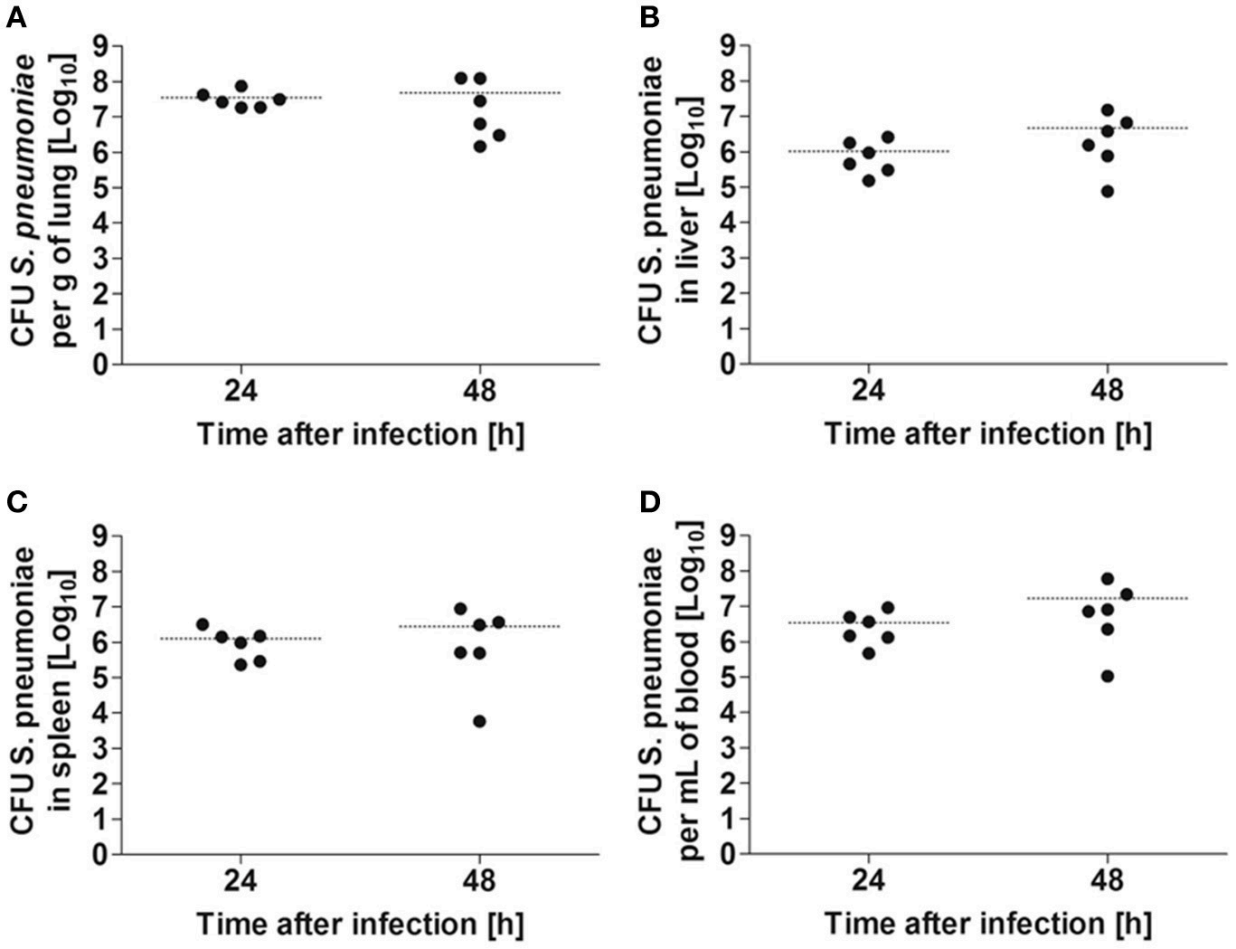
Bacterial burdens in the organs of Balb/c mice after intranasal infection with 5 × 10^7^ CFU of *S. pneumoniae*. Bacterial loads were determined in lung **(A)**, liver **(B)**, spleen **(C)**, and blood **(D)** at 24 and 48 h after bacterial inoculation. Each symbol represents an individual animal. Horizontal lines indicate the mean value. One representative experiment out of three is shown.

Balb/c mice were intranasally inoculated with either 5 × 10^7^ CFU of *S. pneumoniae* TIGR4 or with PBS as a mock treatment, and disease progression was evaluated by body weight loss, granulocyte/lymphocyte ratio in blood and bacterial loads in the lungs, blood, and systemic organs. Significant body weight loss was already observed in *S. pneumoniae*-infected mice 24 h after inoculation compared to PBS-treated animals, being even more pronounced at 48 h after treatment (Figure 1A). This was accompanied by a significant increase of the blood granulocyte/lymphocyte ratio at 48 h indicating acute inflammation (Figure 1B). The highest bacterial loads of *S. pneumoniae* were observed in the lungs. The bacteria of *S. pneumonia-*infected mice were found to have spread from the lungs to the systemic organs such as the liver and spleen and were also present in high loads in the blood leading to bacteremia (Figure 2). These results are expected for a highly virulent strain as it has been previously demonstrated that the number of bacteria in the blood increases rapidly during the first 3 h after challenging with *S. pneumoniae* serotype 4 (Aaberge et al., 1995).

### Comparative proteomic analysis of the lung by DIGE

**Figure 3:**
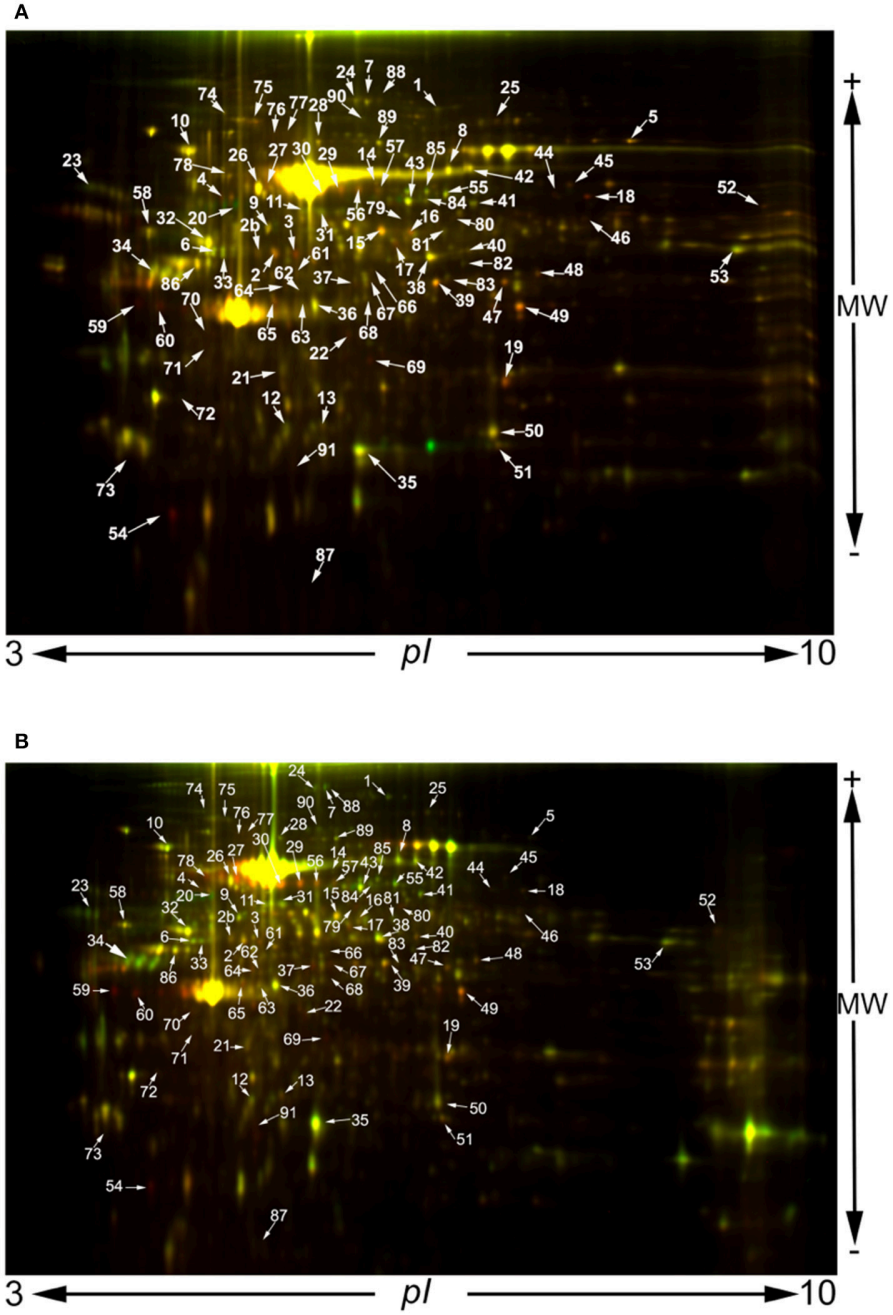
2D-DIGE images obtained at 24 h **(A)** and 48 h post infection **(B)**. A protein concentration of 50 μg of each sample was minimally labeled with 400 pmol CyDyes DIGE Fluors: Cy3 for PBS controls and Cy5 for *S. pneumoniae* TIGR4-infected samples and vice versa to reduce variation due to labeling. An internal standard was generated by combining equal amounts of extracts from all the samples generated at 24 or 48 h, respectively, and labeled with Cy2.

In order to investigate the effect of *S. pneumoniae* infection on lung tissue at a molecular level we compared *S. pneumoniae*-infected lungs with those of PBS-treated control mice. We analyzed two time points: 24 and 48 h. For each time point we used three biological replicas and in each biological replica four technical replicas were performed. Although the production of such a large number of DIGE gels and the image analysis are very laborious, this experimental design provides reliable robust results. Using this approach, mass spectrometry identified 91 spots from lung tissue, the abundance of which was modified during infection by *S. pneumoniae* (see Figure 3 for a representative coloured images of 2DE maps, as well as Figure 1S where all the replicas used for the analysis are shown). M_r_ and p*I* of protein spots were experimentally determined and compared with gene-deduced M_r_ /p*I* coordinates obtained from MASCOT (Table 1S). The majority of gel-estimated and theoretical M_r_/p*I* fit quite well. Lower M_r_ values could be due to post-translational processing or proteolysis, while higher M_r_ values could be the result of covalent binding of chemical groups.

Mucus plays a key role in protecting against bacterial colonization of the epithelia. During lung infection the production of mucus and also mucociliary flow are increased in order to mechanically clear bacteria. Dynein is a family of cytoskeletal motor proteins which transports various cellular cargos, provides forces and displacements which are important for mitosis and drives the beating of eukaryotic cilia and flagella (Lindemann, 1994). One of the responses to respiratory pathogens involves mucus secretion and clearance by ciliary motility (Mckenzie et al., 2013). However, electron microscopy has shown that pneumolysin, a key virulence factor of *S. pneumoniae*, causes ciliary slowing (Steinfort et al., 1989). In the present study we identified two dyneins: axonemal dynein heavy chain 8 long form (Spot 6) and dynein heavy chain 2 (Spot 37). The abundance of the first dynein was reduced at both time points, while that of the second increased at both time points. Dynein heavy chain 2 has a specialized role in transporting material along motile and sensory cilia and flagella, and dynein heavy chain 8 is built into the axoneme where it powers ciliary beating. A reduction in axonemal dynein heavy chain 8 long form (Spot 6) abundance could explain ciliary slowing. It is worth mentioning that dyneins are large proteins of about 500 kDa, which cannot be resolved in 2-D gel electrophoresis due to technical limitations. Surprisingly, however, we detected both proteins with a lower molecular mass, probably due to proteolysis events. Nonetheless, these proteolized forms were present in both control and infected lungs. The presence of these proteolized forms is probably the result of the normal protein turnover of these proteins, which would suggest the presence of changes in the turnover balance of these proteins during pneumococcal infection.

We also found modification in the abundance of proteins involved in blood coagulation (spots 2, 2b, 15, and 81). It has been proposed that fibrin prevents infection-stimulation blood loss, thereby performing a protective function that is essential for survival. Remarkably, fibrin does not simply protect against vascular damage caused directly by the infectious agent, but rather, protects against hemorrhage evoked by interferon-γ a critical mediator of type 1 immunity (Johnson et al., 2003). In addition to fibrin, we observed a reduction in the abundance of plasminogen (spot 24). Plasmin dissolves the fibrin of blood clots and acts as a proteolytic factor in a variety of cellular processes, including inflammation(Castellino and Ploplis, 2005), and it has been demonstrated that in association with the surface of a pathogen, the host plasminogen can facilitate invasion by mediating tissue barrier degradation and dissemination of the invading organism. Thus, a reduction in the levels of plasminogen abundance would contribute to reducing the spread of the pathogen (Sun et al., 2004).

Interestingly we found that the abundance of the protein encoded by the *Sec14l3* gene was different during *S. pneumoniae* infection. The *Sec14l3* gene encodes a 45-kDa secretory protein, the synthesis of which take places specifically in the airway epithelium. In this sense, Shan et al. found an inverse relationship between *Sec14l3* mRNA/protein expression using the bronchoalveolar lavage fluid in an animal model showing many features similar to human allergic asthma (Shan et al., 2009). In their study using 2DE stained with SYPRO Ruby, the authors observed a down-regulation of the protein following inflammation. In addition, Shan et al. (2012) showed that ciliated cells in mouse airway epithelium selectively express the *Sec14l3* transcript. Using real-time quantitative polymerase chain reaction (rt-PCR), these authors demonstrated that the expression of Sec14l3 transcript is restricted to mouse trachea and lung. Moreover, the same results were obtained in the rat homolog of the Sec14l3 transcript (Merkulova et al., 1999). In rats the Sec14l3 transcript is expressed on the surface layers of the trachea and in the ciliated bronchial epithelium in the lung. In a three-dimensional culture of mouse tracheal epithelial cells, Shan et al. (2012) proposed that levels of the Sec14l3 mRNA were correlated with the differentiation of ciliated cells. In addition, intranasal infection of adult mice with influenza virus resulted in a progressive 20-fold decrease in Sec14l3 mRNA expression over 10 days post infection. In the present study we found that levels of the protein produced by Sec14l3 are modified during *S. pneumoniae* infection. At 24 h post infection this protein showed a 3-fold increase in abundance, while at 48 h the abundance of this protein was reduced −1.2-fold compared to the control. This suggests that the Sec14l3 protein may have a role during the first hours of infection, but when pneumococci invade and cause bacteremia the abundance of this protein is reduced.

The protein lipocortin 1, also known as annexin 1 (Spot 19), plays an important role in innate immune response as an effector of glucocorticoid-mediated responses as well as a regulator of the inflammatory process. It is a 37-kDa protein that is implicated in the control of cell growth and differentiation, signal transduction and arachidonic acid release, in addition to intracellular vesicle trafficking. Furthermore, it has also been postulated that lipocortin 1 is a mediator of glucocorticoid action in inflammation and in the control of anterior pituitary hormone release (Hannon et al., 2002). In the present comparative proteomic analysis we identified two different forms of this protein, both with the same isoelectric point but with a different molecular mass. In the samples collected at 24 h spot 19 showed its predicted molecular weight (37 kDa) and a 2-fold change, and at 48 h the spot also showed an increased 1.7-fold change. Spot 50, also identified as lipocortin 1, has an estimated molecular weight of 29.8 kDa and a 1.4-fold change at both 24 and 48 h. These results suggest that this protein undergoes several processes during *S. pneumoniae* infection. In addition to lipocortin 1, we also observed an increase in thymidylate kinase (spot 67) at both time points. It has been demonstrated that the expression of this enzyme is induced by the presence of lipopolysaccharides (LPS) of pathogenic bacteria (Lee and O'brien, 1995) as well as during bacterial infection (Sharif et al., 2013), and several studies have described thymidylate kinase as a marker for the diagnosis, control of treatment, and follow-up of malignant diseases (O'neill et al., 1992; Look et al., 1997; Xiang et al., 2013), including lung tumors (Korkmaz et al., 2013).

Nearly all bacteria, including opportunistic pathogens of the airway, require an iron source for survival (Weinberg, 2009). Proteins related to iron homeostasis are also affected during *S. pneumoniae* infection. On one hand, the abundance of ferritin (spot 73), which stores iron in a soluble non-toxic and readily available form, decreases while, on the other hand, the abundance of hemopexin (spots 26, 29, and 30) increases. Hemopexin binds the heme group and transports it to the liver for breakdown and iron recovery. A reduction in ferritin abundance demonstrates that respiratory colonizers, including pneumococci, obtain iron by directly retrieving iron-containing molecules (Siegel and Weiser, 2015).

Using this proteomic approach we also identified a protein that has been proposed as a biomarker for discriminating normal bronchial epithelium from neoplastic lesions from invasive squamous cell lung cancer (Zeng et al., 2013). This protein is the selenium-binding protein 1 (Spot 14), a member of the selenoprotein family that has been shown to covalently bind selenium (Behne and Kyriakopoulos, 2001; Jeong et al., 2009) and mediate the intracellular transport of selenium (Porat et al., 2000). Recently, Zeng et al. proposed this protein as a biomarker for invasive squamous cell lung cancer (Zeng et al., 2013). These authors found that abundance of selenium-binding protein 1 is reduced during invasive squamous cell lung cancer. However, in the present study, we also found this protein to have lower abundance in *S. pneumoniae*-infected lungs at both time points studied (24 and 48 h). In addition, we also found the abundance of Indolethylamine N-methyltransferase (Spot 35) to be reduced. This protein plays an important role in the detoxification of selenium compounds and has been also proposed as a biomarker for early meningioma progression (Schulten et al., 2016).

The protein Serpina3K (spot 23) has a relevant role in cell processes during infection. This protein is a serine proteinase inhibitor that has shown to down-regulate the key effectors of the Wnt signaling pathway: β-catenin, nonphospho-β-catenin, and low-density lipoprotein receptor-related protein 6. In addition, this protein also plays an important role regulating the reactive oxygen species system and antioxidants (Zhu et al., 2014). The Wnt signaling pathway is an ancient evolutionarily conserved pathway that regulates crucial aspects of cell fate determination, cell migration, cell polarity, neural patterning, and organogenesis (Komiya and Habas, 2008). Inflammatory response must be tightly regulated because uncontrolled inflammation may lead to tissue injury. Among the many signaling pathways activated, the canonical Wnt/β-catenin pathway plays an important role in the expression of several inflammatory molecules during bacterial infections. The evidence accumulated so far has shown that activation of the Wnt/β-catenin pathway reduces several molecular inflammatory processes triggered by bacterial pathogens (Silva-Garcia et al., 2014). In the present study we found that this protein is less abundant (−1.6-fold change) 24 h post infection, thereby allowing the activation of the Wnt pathway, with a −2.2-fold change at 48 h post infection. Serpinb1A (Spot 22), also known as leukocyte elastase inhibitor A, is another serpin presenting a modification in abundance during *S. pneumonaie* infection. The abundance of this protein is increased at both 24 and 48 h. This protein has shown to play a key role in preserving lung defense functions in different infection models (Benarafa et al., 2007, 2011; Gong et al., 2011).

The GTPase superfamily includes a diversity of molecules the functions of which are regulated through the binding and hydrolysis of guanosine-5′-triphosphate. The expression of one family of putative GTPases has shown to be selectively induced by interferon-gamma (IFN-gamma) and in some cases by IFN-alpha beta or bacterial LPS (Gilly and Wall, 1992; Taylor et al., 1996). This induction pattern implicates these putative GTPases as part of the innate defense of cells to infection. However, their role in this defense has not yet been defined. In the present study we identified two interferon inducible GTPases (Spots 66 and 68) with reduced abundance 24 h post infection, but increased abundance 48 h post infection. Irga6 (Spot 66) has been identified as a necessary factor in conferring host resistance by remodeling a classically nonfusogenic intracellular pathogen to stimulate fusion with autophagosomes, thereby rerouting the intruder to the lysosomal compartment for destruction (Al-Zeer et al., 2009). In addition, Tgtp (Spot 68) has been described to have specific antiviral activity (Carlow et al., 1998), but in the present study we found that the abundance of this GTPase is also increased during *S. pneumoniae* infection.

Among the proteins identified we found several involved in cell cytoskeleton function or reorganization. Electron microscopy has demonstrated that *S. pneumoniae* infection of the lungs produces cytoplasmic blebs, mitochondrial swelling, and cellular extrusion, among other cytoplasmic changes (Steinfort et al., 1989). In addition, neutrophil sequestration is an essential antibacterial defense mechanism in the lung and involves multiple steps, including the activation of transcription factors, the production of chemokines, upregulation of cell adhesion molecules, and enhancement of cell-cell interactions (Craig et al., 2009). Among the cell cytoskeleton-related proteins we identified moesin (Spots 8 and 42). This protein interacts with CD43 (leukosialin, sialophorin). CD43 is able to induce the switching of T lymphocytes from a spherical to a polarized motile morphology, with the formation of a uropod at the rear of the cell (Sperling et al., 1995). It has been shown CD43 plays an important regulatory role in remodeling T-cell morphology through its interaction with actin-binding proteins ezrin and moesin (Serrador et al., 1998). The abundance of these two acting-binding proteins was modified at both time points in our study. Moesin is involved in the formation of filopodia and microvilli-like structures. These are slender cytoplasmic projections that extend beyond the leading edge of lamellipodia and they function as antennae for cells to probe their environment (Mattila and Lappalainen, 2008). It has also been demonstrated that the intracellular bacteria *Ehrlichia* are transported between cells through the host cell filopodia induced by the pathogen during the initial steps of infection (Thomas et al., 2010). In addition, microvilli-like structures are associated with the internalization of virulent capsulated *Neisseria meningitidis* into vascular endothelial cells (Eugène et al., 2002). Furthermore, desmoplakin, a key protein for desmosome formation, is also increased while ezrin, a protein required for macropinocytosis, is decreased. In summary, the abundance of several proteins involved in cell shape, cell-cell interaction, cytoskeleton organization, and vesicle intracellular transport is modified. It is worth mentioning that some of these proteins have a high molecular weight outside the range of proteins suitable for 2-D electrophoresis. However, in the present study we found these proteins to have a low molecular weight compared with the predicted molecular weight. This suggests a great reorganization of cytoskeleton structures related to the transport of vesicles as well as pathogens through the cell in addition to cell-cell interactions.

### Protein relationships

The output of a proteome analysis either in a shotgun approach or as in the present study with a more targeted method, usually includes a long list of the factors identified which are associated with a probability score and ideally also a quantitative value. In order to understand and interpret the results of the systemic response of the proteome to a challenge, the list must be classified and filtered. The first problem is the assignation of an identifier to each protein. While gene names have been standardized, protein names can differ between different databases and even within the same database. In order to avoid confusion in the names/entries of the proteins we looked for the gene names of each entry identified in this work. This information was obtained from the Uniprot knowledge database.

**Figure 4:**
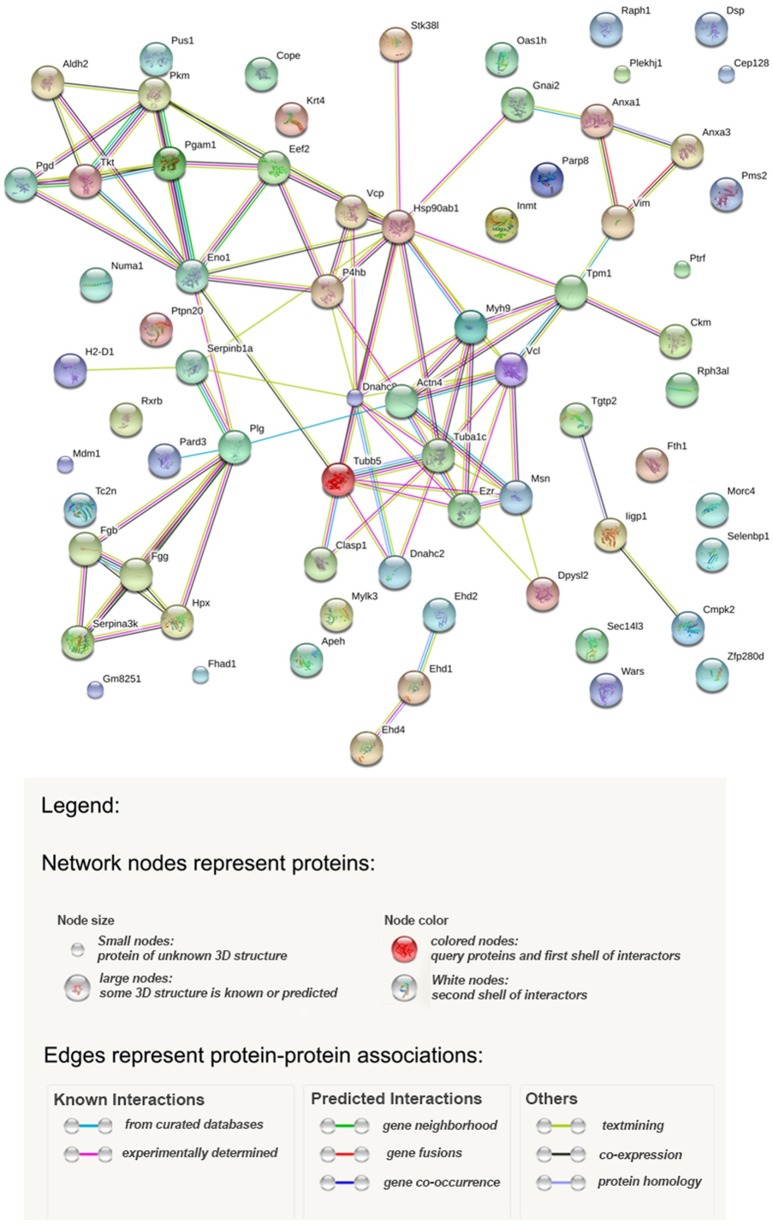
Interaction map of proteins identified as calculated by STRING.

Potential relationships among the proteins identified were analyzed with the STRING database of known and predicted protein-protein interactions which include direct (physical) and indirect (functional) associations (Szklarczyk et al., 2015). Figure 4 shows the results, and Table 2S provides statistical details of the relationships. According to the STRING analysis the most enriched processes from the data obtained were the regulation of vesicle-mediated transport (GO.0060627), wound healing (GO.0042060), and cytoskeleton organization (GO.0007010). These Gene Ontology entries had the best false discovery rate, although response to stress (GO.0006950) showed the greatest gene count with a total of 20 genes. From a functional point of view the most enriched functions were anion binding (GO.0043168), nucleoside binding (GO.0001882), purine ribonucleoside triphosphate binding (GO.0035639), organic cyclic compound binding (GO.0097159), carbohydrate derivative binding (GO.0097367) and heterocyclic compound binding (GO.1901363). Figure 4 shows three clearly represented nodes. The first of which is the fibrinogen system which is involved in wound healing. Secondly, in the middle of the interaction map, there are all the cytoskeleton proteins involved in vesicle-mediated transport as well as cytoskeleton organization. Finally, a set of proteins, mainly metabolic enzymes, are shown interacting with enolase. The presence of these proteins can be explained by the different energy requirements by the cells during infection.

In conclusion, in the present study we used a proteomic approach to investigate lung injury in a mouse model of *S. pneumoniae* infection. This approach revealed a number of important changes in the lung cells, mainly associated with cytoskeleton organization, vesicle-mediated transport, and wound healing. In addition, we observed changes in the abundance of some proteins previously proposed as biomarkers for diseases other than infection by *S. pneumoniae*.

Our results contribute to providing better knowledge of the pathogenesis of respiratory infection caused by *S. pneumoniae* and in particular the effect of these bacteria on alterations in lung tissue.

## Ethics statement

The animal studies in this study were performed in strict accordance with the German regulations of the Society for Laboratory Animal Science (GVSOLAS) and the European Health Law of the Federation of Laboratory Animal Science Associations (FELASA). All experiments were approved by the Ethical Board Niedersächsisches Landesamt für Verbraucherschutz und Lebensmittelsicherheit, Oldenburg in Germany (Permit No. 33.9-42502-04-13/1260).

## Author contributions

MF-N did all experiments concerning proteomic analysis, designed the experiment, and write the paper. AS and EM performed all the animal experiments. JV designed the experiments and write the paper.

### Conflict of interest statement

The authors declare that the research was conducted in the absence of any commercial or financial relationships that could be construed as a potential conflict of interest.

## Supplementary material

Table 1S List of proteins identified.

Table 2S Details of the relationships among the proteins identified according to STRING analysis.

Figure 1S Silver stained images of all the replicas used in this study. The mass spectrometry proteomics data have been deposited in the ProteomeXchange Consortium via the PRIDE (Vizcaino et al., 2016) partner repository with the dataset identifier PXD006393 and 10.6019/PXD006393. (https://www.ebi.ac.uk/pride/archive/projects/PXD006393; username: reviewer12518@ebi.ac.uk; password: OWNrkahJ).
