# Supplementary figures and images for: Changed Expression of Cytoskeleton Proteins During Lung Injury in a Mouse Model of Streptococcus pneumoniae Infection
Source: Front Microbiol. 2018 May 8;9:928. doi: 10.3389/fmicb.2018.00928 (PMC5952171; doi:10.3389/fmicb.2018.00928)

## **Mouse 1 - Gel 1**

**Cy2 - 24 hours**

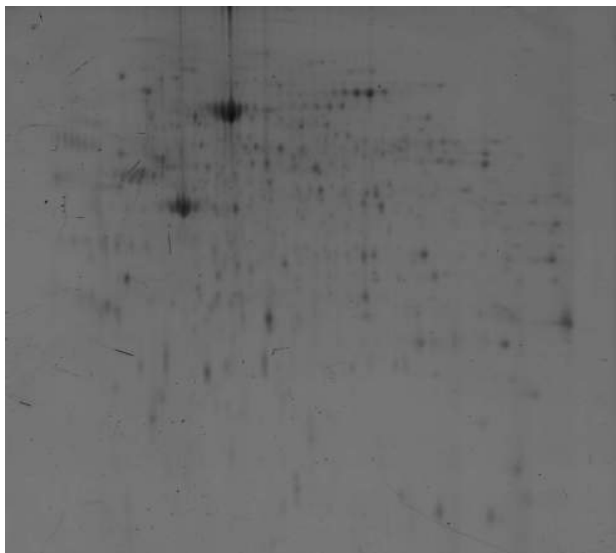

**Cy2 - 48 hours**

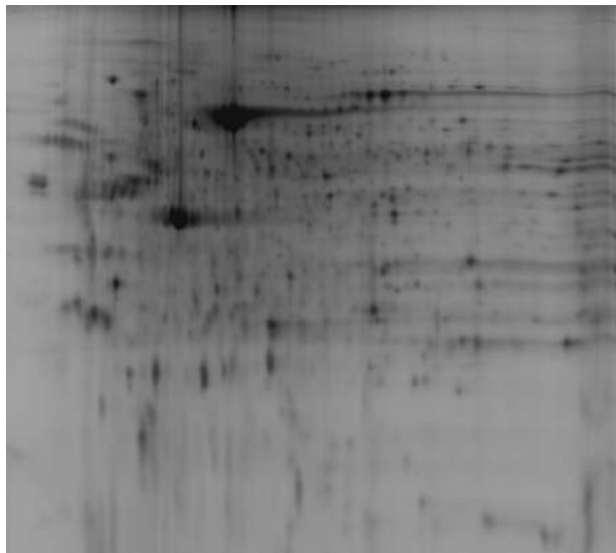

## **Mouse 1 - Gel 2**

**Cy2 - 24 hours**

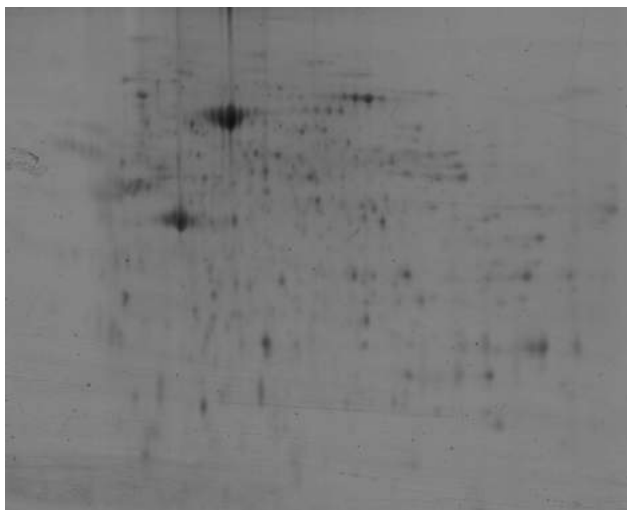

**Cy2 - 48 hours**

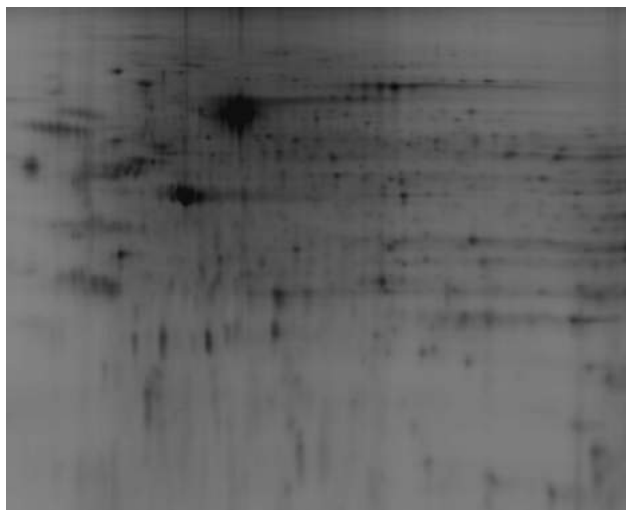

Supplement: Supplementary file 4 [file Presentation_2.pdf]

## Mouse 2 Gel 1

Cy2 - 24 hours

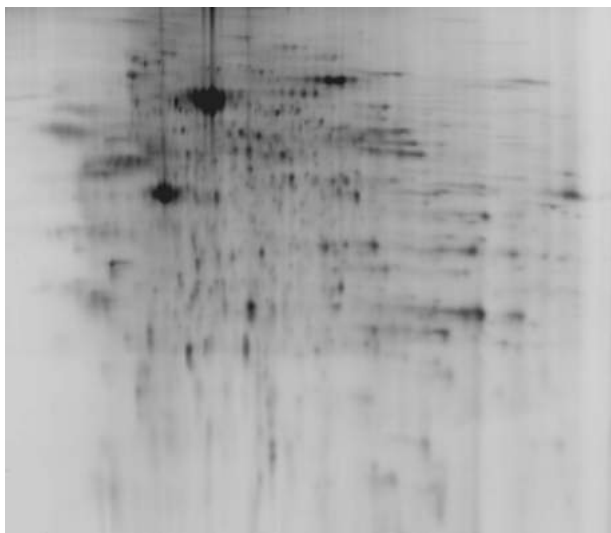

Cy2 - 48 hours

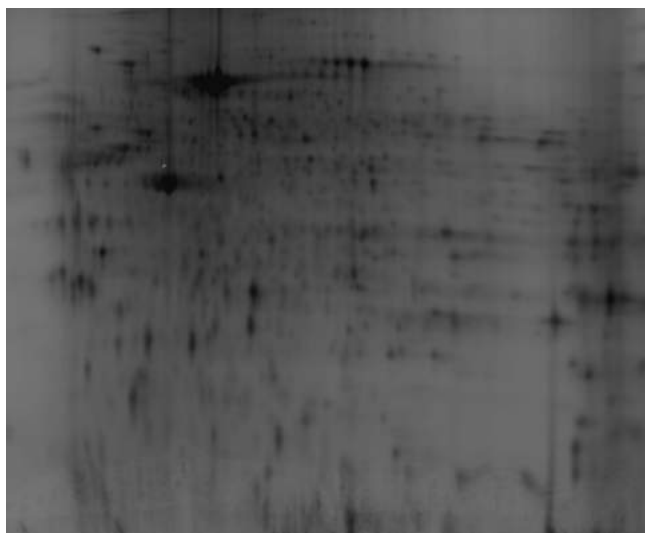

## Mouse 2 Gel 2

Cy2 - 24 hours

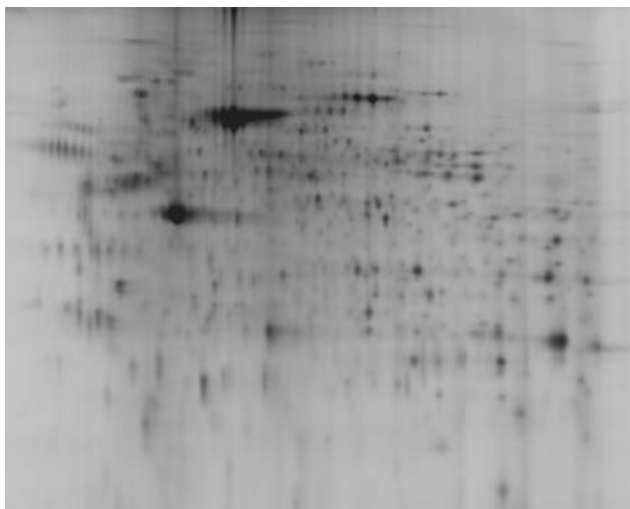

Cy2 - 48 hours

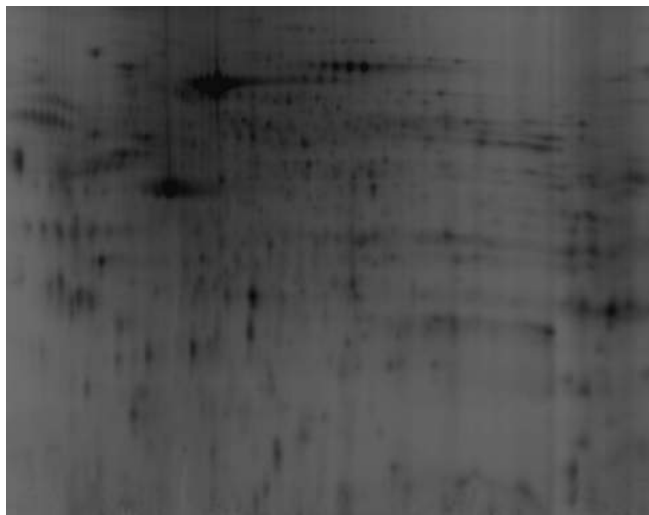

Supplement: Supplementary file 5 [file Presentation_3.pdf]

## Mouse 2 Gel 3

Cy2 - 24 hours

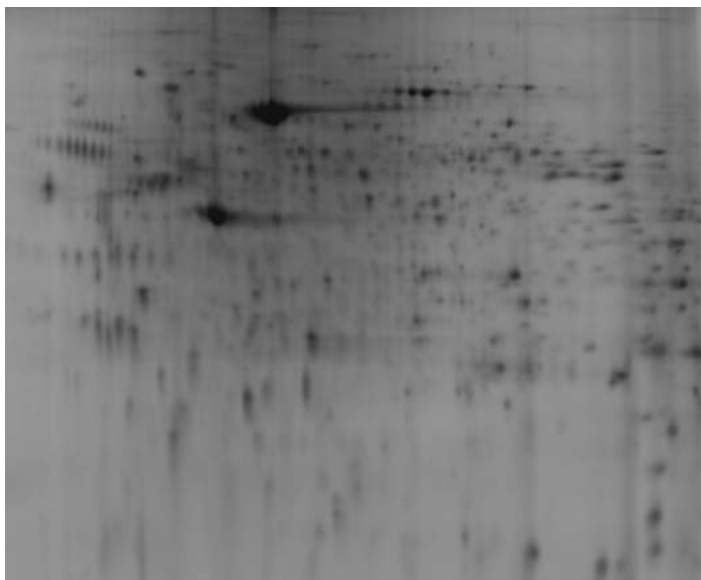

Cy2 - 48 hours

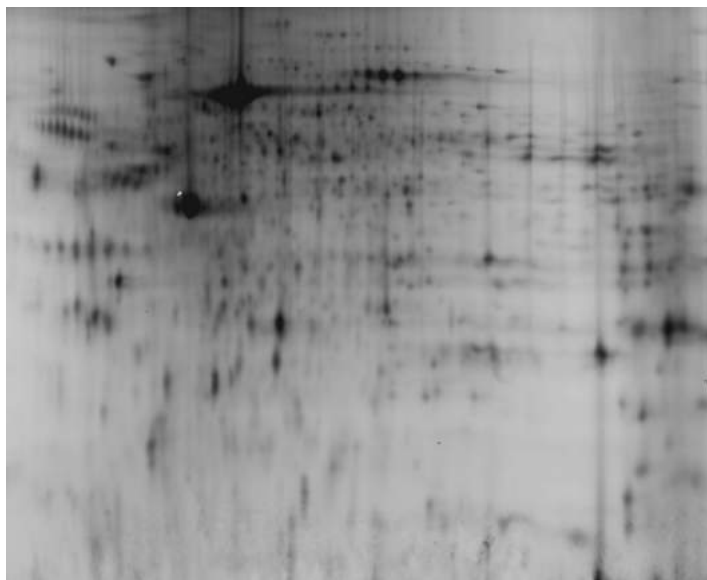

## Mouse 2 Gel 4

Cy2 - 24 hours

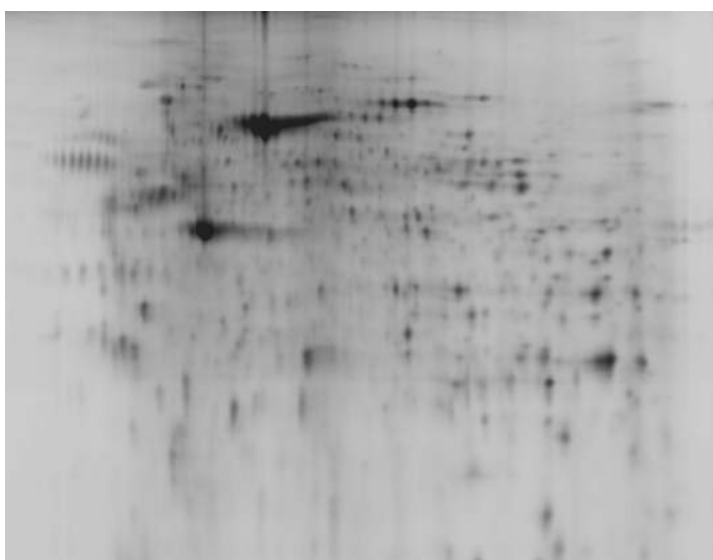

Cy2 - 48 hours

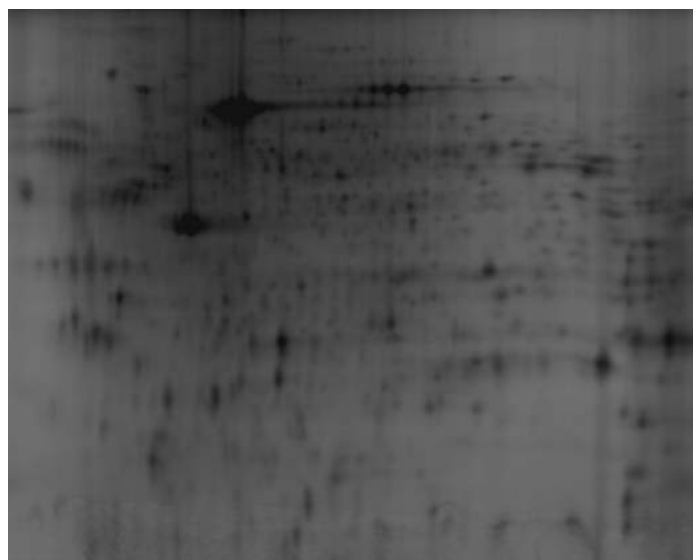

Supplement: Supplementary file 6 [file Presentation_4.pdf]

## Mouse 3 Gel 1

Cy2 - 24 hours

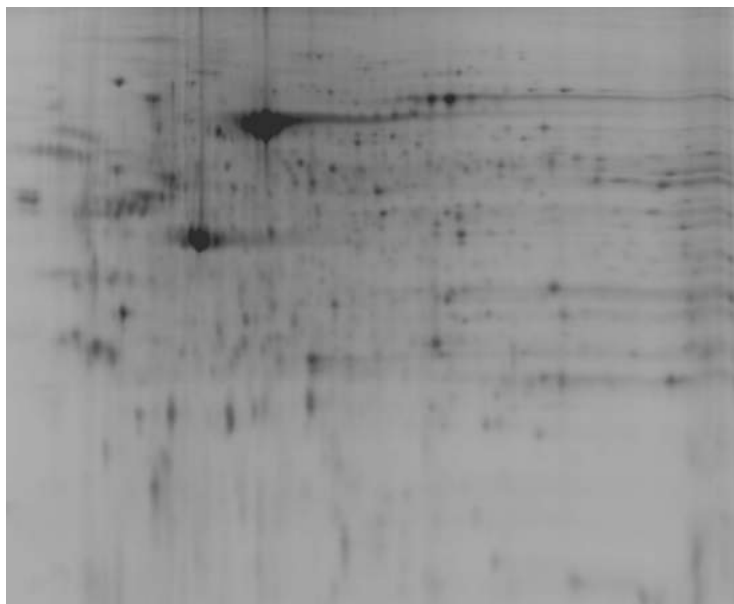

Cy2 - 48 hours

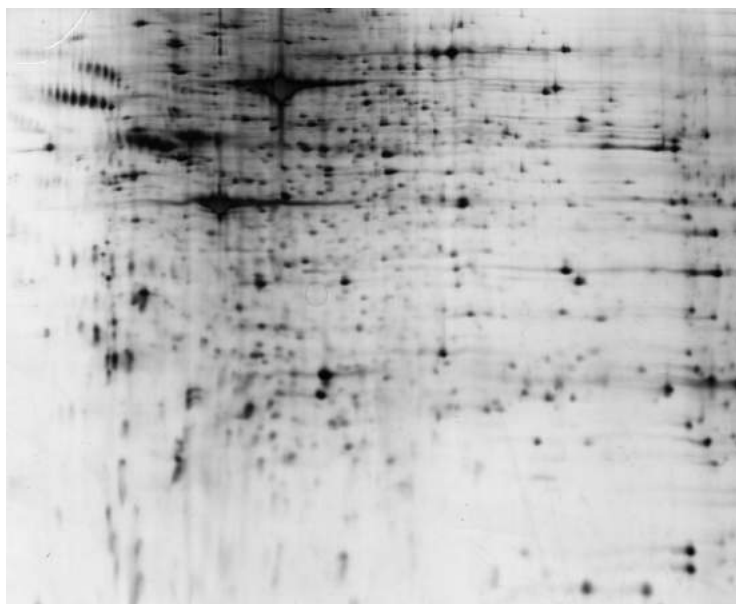

## Mouse 3 Gel 2

Cy2 - 24 hours

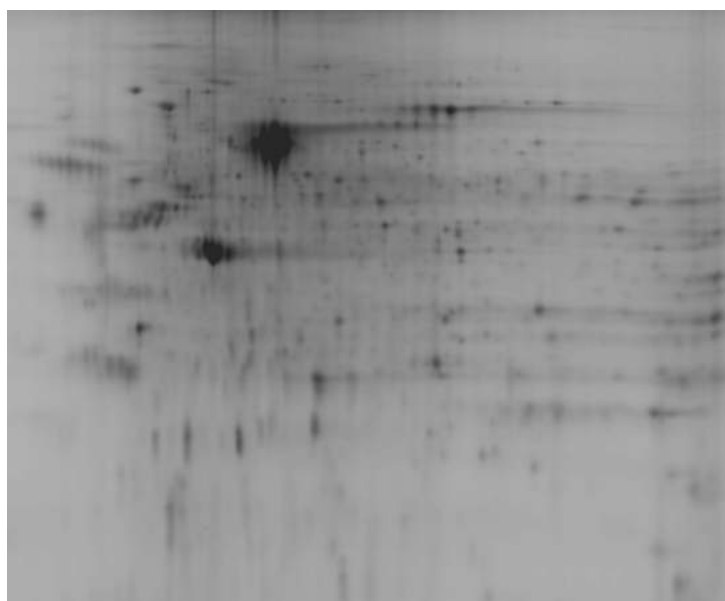

Cy2 - 48 hours

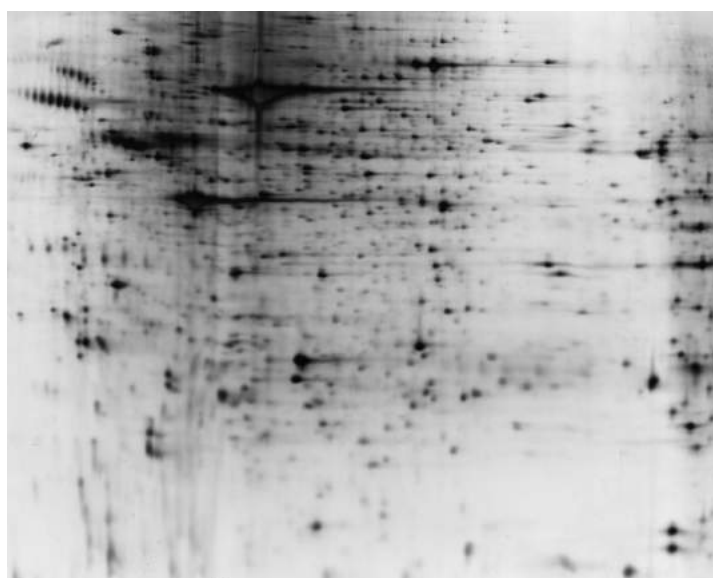

Supplement: Supplementary file 7 [file Presentation_5.pdf]

### **Mouse 3 Gel 3**

**Cy2 - 24 hours**

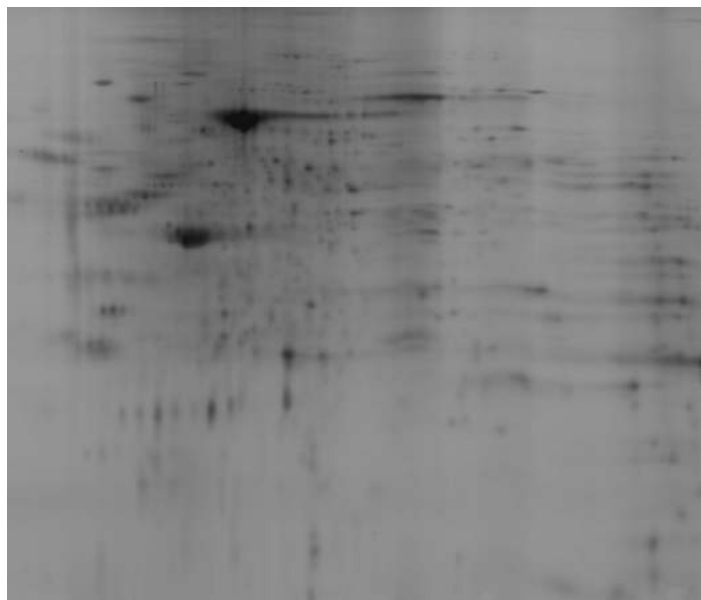

**Cy2 - 48 hours**

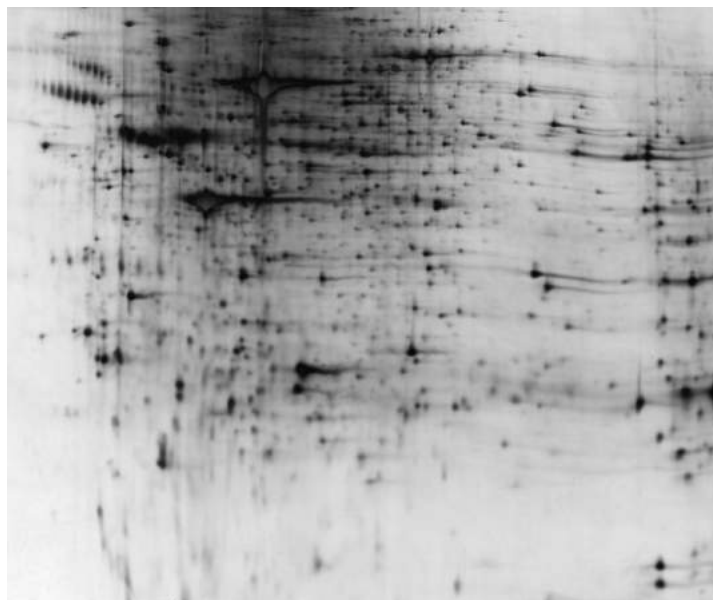

### **Mouse 3 Gel 4**

**Cy2 - 24 hours**

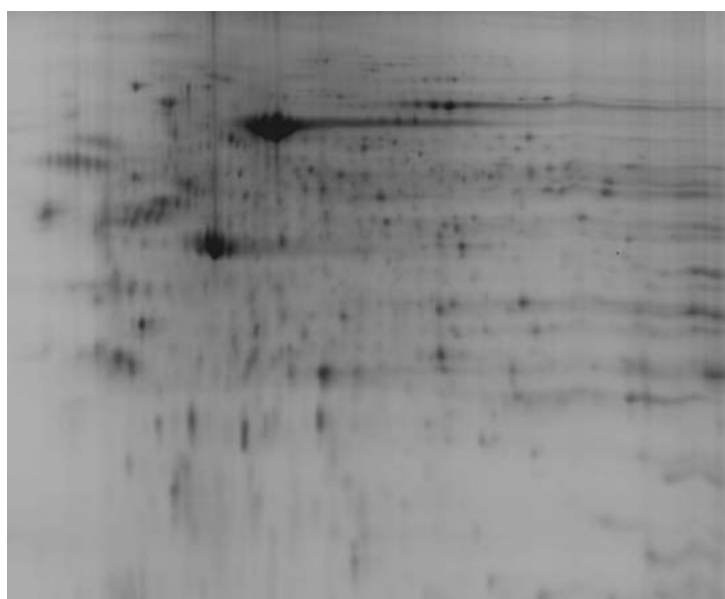

**Cy2 - 48 hours**

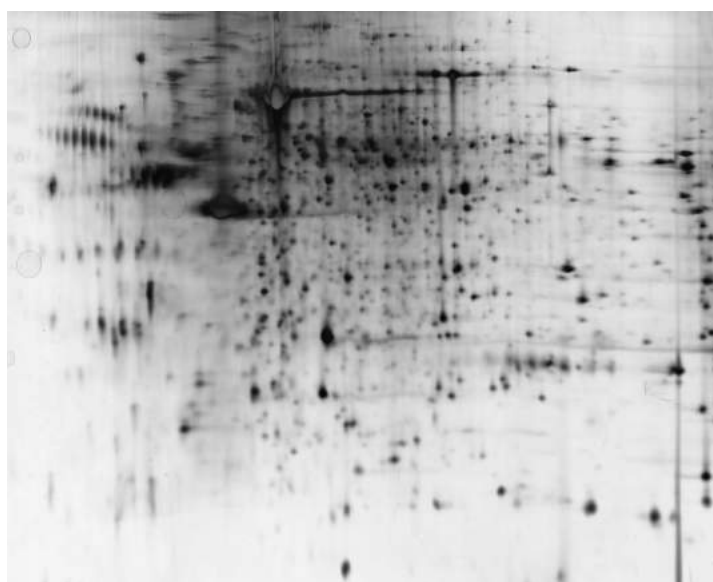

Supplement: Supplementary file 8 [file Presentation_6.pdf]
