# Supplementary material for: Changed Expression of Cytoskeleton Proteins During Lung Injury in a Mouse Model of Streptococcus pneumoniae Infection
Source: Front Microbiol. 2018 May 8;9:928. doi: 10.3389/fmicb.2018.00928 (PMC5952171; doi:10.3389/fmicb.2018.00928)

Mouse 1 Gel 3

Cy2 - 24 hours

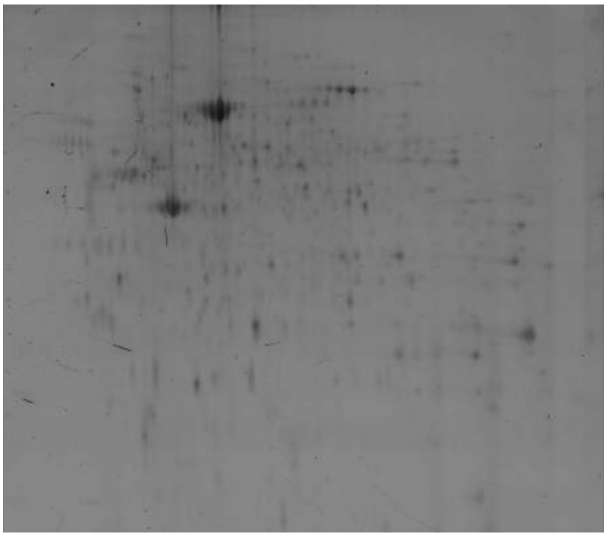

Cy2 - 48 hours

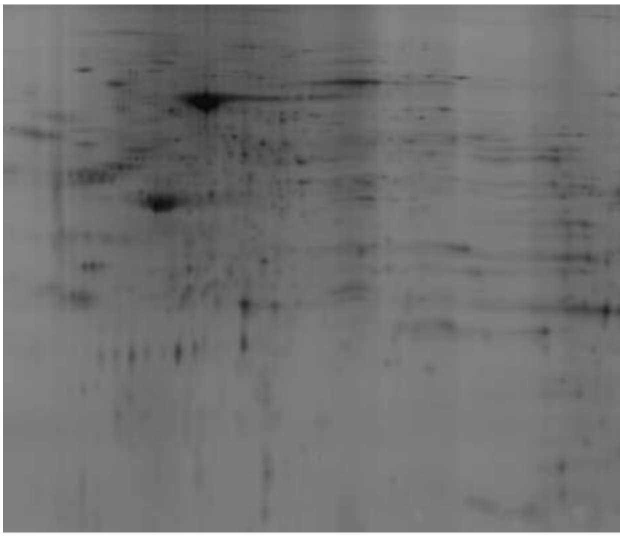

Mouse 1 Gel 4

Cy2 - 24 hours

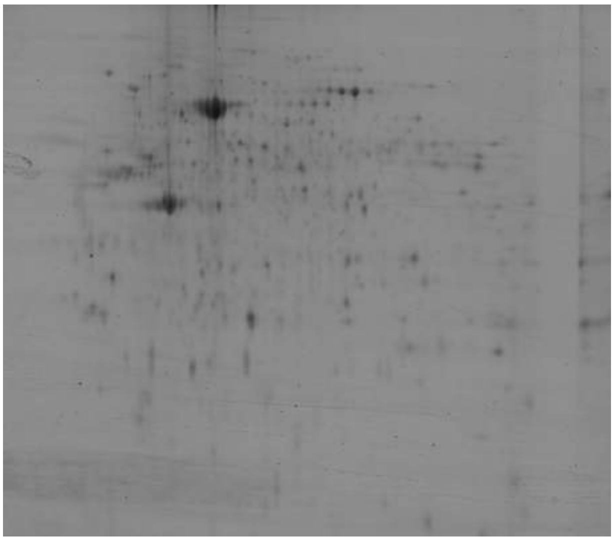

Cy2 - 48 hours

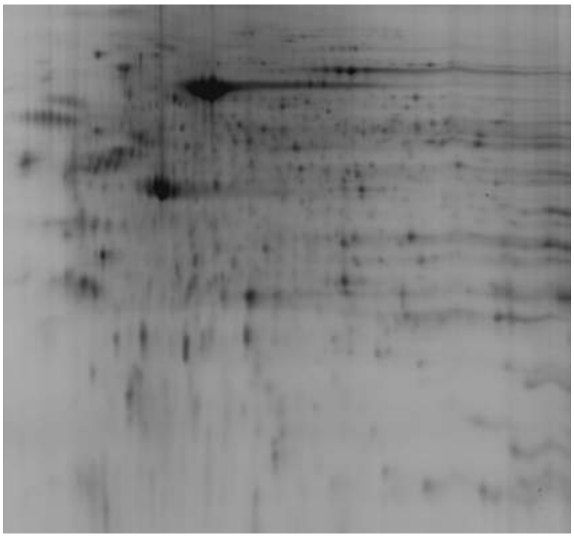

Supplement: Figure 1S — Silver stained images of all the replicas used in this study. The mass spectrometry proteomics data have been deposited in the ProteomeXchange Consortium via the PRIDE (Vizcaino et al., 2016) partner repository with the dataset identifier PXD006393 and 10.6019/PXD006393. (https://www.ebi.ac.uk/pride/archive/projects/PXD006393; username: reviewer12518@ebi.ac.uk; password: OWNrkahJ). [file Presentation_1.pdf]
